# The New Status of Parasitic Diseases in the COVID-19 Pandemic—Risk Factors or Protective Agents?

**DOI:** 10.3390/jcm10112533

**Published:** 2021-06-07

**Authors:** Kinga Głuchowska, Tomasz Dzieciątkowski, Aleksandra Sędzikowska, Anna Zawistowska-Deniziak, Daniel Młocicki

**Affiliations:** 1 Department of General Biology and Parasitology, Medical University of Warsaw, 02-004 Warsaw, Poland; kinga.gluchowska@wum.edu.pl (K.G.); aleksandra.sedzikowska@wum.edu.pl (A.S.); 2 Chair and Department of Medical Microbiology, Medical University of Warsaw, 02-004 Warsaw, Poland; tomasz.dzieciatkowski@wum.edu.pl; 3 Witold Stefański Institute of Parasitology, Polish Academy of Sciences, 00-818 Warsaw, Poland; anna.zawistowska@twarda.pan.pl

**Keywords:** COVID-19, SARS-CoV-2, parasites, diseases, parasitosis, pandemic

## Abstract

It is possible that parasites may influence the course of COVID-19 infection, as either risk factors or protective agents; as such, the current coronavirus pandemic may affect the diagnosis and prevention of parasitic disease, and its elimination programs. The present review highlights the similarity between the symptoms of human parasitoses and those of COVID-19 and discuss their mutual influence. The study evaluated selected human parasitoses with similar symptoms to COVID-19 and examined their potential influence on SARS-CoV-2 virus invasion. The available data suggest that at least several human parasitoses could result in misdiagnosis of COVID-19. Some disorders, such as malaria, schistosomiasis and soil-transmitted helminths, can increase the risk of severe infection with COVID-19. It is also suggested that recovery from parasitic disease can enhance the immune system and protect from COVID-19 infection. In addition, the COVID-19 pandemic has affected parasitic disease elimination programs in endemic regions and influenced the number of diagnoses of human parasitoses.

## 1. Introduction

Various infectious agents may simultaneously and independently invade the human body. As microorganisms and parasites share mechanisms of pathogenesis, eliciting similar inflammation processes, immune or allergic reactions, it is plausible that co-infections may lead to misdiagnosis and false estimates of the real prevalence of single infective agents. Such co-existence may also lead to a more severe course of infection.

The appearance of new infectious agents presents a challenge for both the health care system and researchers trying to predict their long-term epidemiological and health consequences; such agents are typically accompanied by new risk factors, contributing to a more severe course and requiring new diagnostic approaches. One such recently arrived threat is the coronavirus infection, as evidenced by the recent COVID-19 pandemic.

COVID-19, caused by the SARS-CoV-2 virus (Coronaviridae), is the infectious agent responsible for the current pandemic. Since the first cases were identified in Wuhan, China, on 17 November 2019, the infection spread rapidly, and was declared a pandemic on 11 March 2020. Since then, the pandemic has continued unabated, with considerable effects on public health, lifestyle and the global economy.

**Figure 1:**
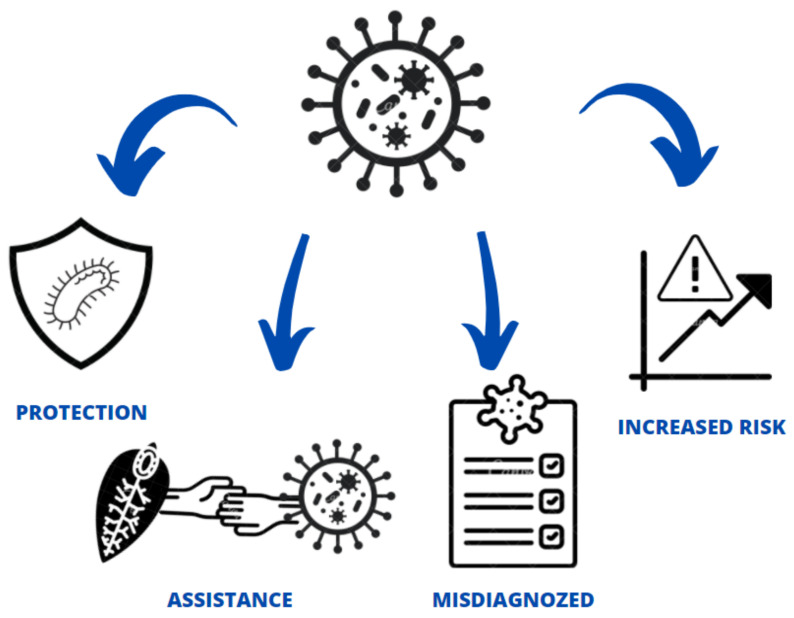
Possible impact of parasites on COVID-19 infection in humans.

As infection is characterized by fever with cough and dyspnea, it can be easily mistaken for other respiratory system diseases, most commonly influenza. Interestingly, similar symptoms are observed in a number of parasitoses, and although these are most commonly associated with populations inhabiting poorer regions, they are also found in richer countries [1]. Not only can parasite infections influence the immune system, destroy tissue, cause anemia and malnutrition, they can also potentially support virus infection [2,3] and affect the effectiveness of vaccines [4,5]. However, parasitic immunomodulation may also protect from tissue damage by reducing inflammatory processes [6]. The present study therefore discusses how parasites may act as risk factors or protective agents in the COVID-19 pandemic and, conversely, how the pandemic may affect the diagnosis and prevention of parasitic diseases (Figure 1).

## 2. COVID-19 Symptoms

Coronavirus Disease 2019 (COVID-19) is an acute infectious disease of the respiratory system caused by the coronavirus SARS-CoV-2. Coronaviruses are relatively large, enveloped viruses that can infect a range of mammals by genetic recombination and variation. SARS-CoV-2 mainly attacks the respiratory system, causing flu-like symptoms such as fever, coughing and asthenia [7]. These symptoms are sometimes accompanied by loss of taste or smell. In children, SARS-CoV-2 infection has also been found to elicit various digestive symptoms, including nausea, vomiting, diarrhea and stomachache [8]; however, most adult patients present with respiratory and digestive symptoms, and slightly fewer with respiratory symptoms alone [9]. A study of 1099 COVID-19 patients showed lymphocytopenia in 83% of blood samples, thrombocytopenia in 36% and leucopenia in 34% [10].

Although infection is mild or asymptomatic in about 60–80% of cases, it takes a severe course in around 5%, particularly among older patients or those with immunodeficiencies. In such cases, there is a high probability of respiratory failure, pneumonia, shock and multiorgan failure; in the most serious cases, infection can be fatal, predominantly due to progression to ARDS and multiorgan failure [11].

## 3. Comparison with Symptoms of Selected Parasitoses

**Figure 2:**
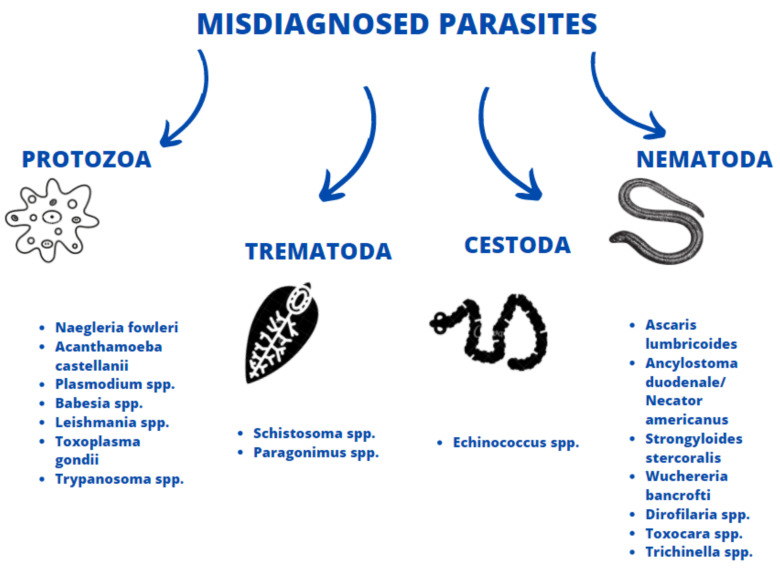
Species of parasites with symptoms mimicking COVID-19.

The general symptoms of COVID-19 may resemble a number of other pulmonary symptoms and diseases, some of which can be caused by parasites. Parasitic pneumonia mostly occurs during larval migrations, when parasites pass through the lungs, but it has been observed to arise as a direct extension from contiguous sites, or due to sequestration of the parasite in the pulmonary capillaries [12]. Such cases are manifested as coughing, fever, breathlessness, rapid breathing and, in severe cases Löffler’s syndrome, due to the antigenic reaction against the parasites or the mechanical rupture of alveoli. Moreover, similarly to SARS-CoV-2, intestinal parasites may cause various digestive symptoms, including nausea, vomiting, diarrhea and abdominal pain. The number of soil-transmitted helminth (STH) cases and their geographic distribution significantly differ from the number and geographic distribution of malaria cases, human echinococcosis or paragonimiasis. Therefore, the epidemic status and the clinical significance of human parasitoses varies and should be taken into account depending on the region of occurrence. General similarities and differences between selected parasitic disorders and SARS-CoV-2 infection are indicated below (Figure 2).

### 3.1. Protozoans

Numerous protozoans, such as malaria and trypanosomiasis, as well as lesser known and rarely diagnosed species, such as amoebae, are regarded as priorities by the WHO. Selected free-living species can occasionally infect people [13], with a mortality rate of up to 99%. The most commonly observed symptom is fever, related to the immune response; however, patients with *Naegleria fowleri* or *Acanthamoeba castellanii* infection also report loss of smell and taste, which are characteristic of SARS-CoV-2 infection [14]. Although the respiratory symptoms are relatively rare, infection increases the chance of co-infection.

*N. fowleri* are generally found in warm freshwater sources. Upon entering the body, typically through the nostrils, the parasite enters the central nervous system (CNS) and moves to the brain through the olfactory nerve and begins destroying the nerve tissue [15]. Naegleria infection causes hemorrhagic necrosis of the brain, resulting in primary amoebic meningoencephalitis (PAM). The symptoms of PAM are headache, stiff neck, fever (38.5–41 °C), altered mental status, seizures and coma [16]. The secretion of cytokines and exogenous exotoxins can cause headaches and damage to the olfactory nerve occurring through lysis of nerve cells and demyelination may result in the loss of smell sensations; in addition, the increase in intracranial pressure resulting from cerebrospinal fluid accumulation can also stimulate the area postrema to create sensations of nausea. Ultimately, the accumulation of cerebrospinal fluid results in the destruction of the CNS. The disease has a high rate of progression and mortality [17,18].

Granulomatous amoebic encephalitis (GAE) is a disease caused by *A. castellanii* [14], which predominantly affects patients with impaired immunity, malignant tumors, diabetes and chronic steroid medications. Acanthomoeba enters the body through damaged skin or the respiratory system and then moves to the brain through the bloodstream. The clinical symptoms of GAE are not specific, and the effects of Acanthomoeba in the brain are similar to those of Naegleria infection, with headaches, fever, disturbances in taste, smell and vision, hemiplegia, damage to the cranial nerves and ataxia [13,19,20].

Another widespread and highly significant disease affecting humans is malaria. According to WHO in 2019, there were 229 million cases of malaria, resulting in 409,000 deaths. In comparison, by 26 February 2021, the number of COVID-19 cases was 113 million with over 2.5 million deaths.

Malaria is a transmissible disease caused by Plasmodium protozoans and spread by Anopheles mosquitos [21]. All types of Plasmodium elicit similar symptoms upon infection, including fever, chills, sweats, headaches, nausea and vomiting, body aches and general malaise, with the time to onset varying according to the species [22,23]. One of the most serious types is pulmonary malaria, which should be differentiated from COVID-19 in endemic areas. In many cases, bilateral pulmonary infiltrates can be observed on lung X-rays. Cabral et al. described three cases of malaria with sickle cell anemia and pulmonary infiltrates [24] and, in another case, two out of three children in one family presented with bilateral fluffy pulmonary infiltrates [25]. Infection can lead to the breakdown of red blood cells (RBCs) and severe anemia, which may adversely affect the course of COVID-19 comorbidity and hence result in poorer survival. Conversely, it has also been predicted that the COVID-19 pandemic may result in up to 36% more malaria deaths over five years compared with the pre-pandemic situation [26]. In addition, gastrointestinal symptoms, such as abdominal pain and diarrhea, are frequently observed in patients with *Plasmodium falciparum* malaria [22,23].

An important risk factor in patients with malaria and babesiosis is adult respiratory distress syndrome (ARDS). In addition, babesiosis patients also demonstrate fever, headache, loss of appetite, myalgia, tiredness and drenching sweats, especially those with a defective immune system [27,28,29]. They also display low and unstable blood pressure, severe hemolytic anemia and thrombocytopenia, which are also present in COVID-19. The similarity of malaria and COVID-19 symptoms can result in one disease being misdiagnosed with the other, or the possibility that co-infection may be missed [30].

Another serious parasitosis is visceral leishmaniasis. It affects several internal organs, including the spleen and liver, and is caused by infection with *Leishmania donovani, L. chagasi* or *L. infantum* [14]. The initial symptoms, viz. fever, chills and headache, appear after an incubation period ranging from a few weeks to months. The fever itself is more intense in the mornings and evenings, reaching above 40 °C. Later, enlarged lymph nodes develop, together with hepatosplenomegaly, anemia, leukopenia, thrombocytopenia, hypoalbuminemia and hypergammaglobulinemia. Patients may also bleed from their gums and nose and present with hyperpigmentation [31,32].

Another transmissible disease, this time transmitted by the Tsetse fly, is trypanosomiasis, caused by *Trypanosoma* species. Two forms of trypanosomiasis exist: Chagas disease, or American trypanosomiasis, and Human African Trypanosomiasis (HAT) [14]. In American trypanosomiasis, infection is mostly focused on the heart muscle, brain and esophagus, while in HAT, the symptoms are more focused on the respiratory system. In both cases, the first stage of infection is local infiltration of mononuclear cells. After a few weeks or months, the patient experiences fever, up to 40 °C, enlarged lymph nodes and Winterbottom’s sign, and blood tests reveal anemia, hypoalbuminemia and hypergammaglobulinemia [33]. This state develops into strong headache, states of agitation or apathy, disturbed consciousness, sleep disturbances, personality changes and eventually, coma [34,35].

*Toxoplasma gondii* is the globally distributed opportunistic parasite that causes toxoplasmosis. Humans are most commonly infected by eating uncooked meat or products laden with oocysts. Symptomatic toxoplasmosis may co-occur with headache, mild fever, up to 38 °C, weakness, muscle pain, anemia and tiredness. The ‘classic’ sign of infection for adults is a nidus of fluffy white, necrotizing retinitis or retinochoroiditis adjacent to a variably pigmented chorioretinal scar [36]. Toxoplasmosis is particularly burdensome in HIV-infected patients, with infection manifesting mainly as encephalitis, chorioretinitis and pneumonitis, or as disseminated infection, depending upon the immune status of the host. In HIV-infected patients, disseminated toxoplasmosis may occur with fever, sepsis-like syndrome with hypotension, disseminated intravascular coagulation [37], as well as neuropsychiatric disorders such as psychosis, dementia, anxiety and personality disorders [38].

### 3.2. Trematodes

The human respiratory system can be infected by flukes; of these, *Schistosoma* spp. causing schistosomiasis, and *Paragonimus* spp., causing paragonimiasis, demonstrate very similar symptoms to COVID-19.

Most cases of schistosomiasis are asymptomatic or may proceed with only a rash. A study of 60 patients found eight to have pulmonary symptoms, with another eight reporting dry cough, shortness of breath, lymphadenopathy, nausea, vomiting, loose stools (sometimes with blood) and enlarged liver, without concurrent fever; however, some reported nocturnal fever peaks [39]. The chronic infection is characterized by abdominal pain, anemia, enlarged liver and blood in the stools or urine [40]. Nodules are also visible in the lungs: the smaller ones range from 2 to 15 mm in size and the larger nodules have a ground glass-opacity halo [41]. The symptoms of intestinal schistosomiasis, includes diarrhea, abdominal pain, dyspepsia, and malnutrition, and they are non-specific [40].

The pulmonary paragonimiasis is believed to occur in 76–90% of cases of Paragonimus infection [42]; symptoms include coughing up rusty brown or bloodstained sputum and recurrent hemoptysis, resulting in a potential misdiagnosis of tuberculosis [43]. The patients also report chest pain, fever, chest tightness, difficulty in breathing, mild pleural effusion, bronchiectasis, pneumonitis, or bronchopneumonia. Despite its high morbidity rate, pulmonary paragonimiasis has low mortality.

### 3.3. Cestodes

As adults, none of the tapeworm species can parasitize human lung tissue or cause symptoms similar to COVID-19. However, infection with the larval form can result in significant debilitation associated with the invasion of various tissues, including the lungs, and affect the immune system response to infections.

One of the most important helminthic pulmonary diseases is cystic echinococcosis, resulting from infection with *Echinococcus granulosus*. After ingestion of infective eggs, the hexacanth hatches in the intestines and migrates with the blood circulation. It most commonly moves to the liver but has been found to infect the lungs in 20% of cases. The initial symptoms are non-specific: the clinical pulmonary symptoms include coughing with clear sputum, dyspnea, chest pain and fever. In 72% of cases of lung infection, the pulmonary cysts appear only in one lobe. The presence of pulmonary echinococcosis is often not apparent on X-ray imaging but can usually be distinguished on CT scan. The rupture of hydatid cysts may result in expectoration of cystic fluid containing parasite membrane, as well as hemoptysis, asthma-like symptoms, respiratory distress, persistent pneumonia, anaphylactic shock, sepsis, elevation of serum IgG and eosinophilia. Rupture of the cysts into the pleura may result in pleural effusion, empyema, and pneumothorax [27].

### 3.4. Nematodes

Ascariasis is a widely distributed infection of the small intestine caused mainly by *Ascalis lumbricoides* nematodes. The infective larva is released in the human small intestine. From here, it penetrates the intestinal wall and migrates through the bloodstream to the lung alveoli, where they grow and molt [44]. During migration, the patient can experience coughing, fever, breathlessness, rapid breathing, chills, paleness and muscle pain. A very common symptom is Loffler’s syndrome, a self-limiting lung inflammation associated with pulmonary eosinophilia [45]. The migrating larvae can induce tissue-granuloma and lung-granuloma formation with macrophages, neutrophils and eosinophils, resulting in hypersensitivity and peribronchial inflammation, as well as increased bronchial mucus production and bronchospasm [27]. Hypereosinophilia is a major feature of ascariasis allowing to discriminate with COVID-19 associated leucopenia. The chest X-ray may show pulmonary infiltrates [45]. In heavy intestinal ascariasis infestation, a mass of worms can block a portion of your intestine. This can cause severe abdominal cramping and vomiting [45].

The symptoms of human hookworm infections, such as those associated with the migration of *Ancylostoma duodenale* and *Necator americanus* larvae, can be confused with those of COVID-19. In these cases, the larvae penetrate through the skin, enter the bloodstream, and move to the heart and lungs. The typical symptoms elicited by migrating larvae are similar to those of pulmonary ascariasis, comprising Loffler’s syndrome, coughing and expectoration, in addition to symptoms characteristic of bronchitis: fever, muscle aches, joint pain, breakdown, headaches, general weakness, malaise and wheezing. After being coughed up and swallowed, the larvae parasitize the small intestine and feed on blood, causing protein-deficiency or iron-deficiency anemia [46,47], which may result in easier incursion of other infections, such as SARS-CoV-2. The presence of adult parasites in the intestine may result in abdominal pain, colic, intestinal cramps, nausea, blood in stool and a loss of appetite.

Strongyloidiasis is caused by *Strongyloides stercoralis* infection; however, diagnosis should be performed with caution. The parasite invasion follows a similar route to that of hookworm infection; however, the clinical symptoms depend on the level of infection [48] and the presentation varies significantly between cases [49]. Even so, eosinophilia, coughing, fever and symptoms of bronchitis or pneumonitis are typically observed as the larvae migrate to the lungs. Strongyloides infection can be clinically unapparent. The usual gastrointestinal symptoms include nausea, vomiting, constipation, and stomachache. Immunocompromised patients are more likely to present with hyperinfection syndrome, i.e., where autoinfection increases the parasitic burden, and disseminated strongyloidiasis. The disseminated form is characterized by the parasite being found in an atypical location, such as the liver, muscle, heart or central nervous system [50]. Physicians need to be aware that corticosteroid and tocilizumab treatment can facilitate Strongyloides infection, resulting in hyperinfection or disseminated infection. At least two cases of subsequent Strongyloides infection have been recorded in patients with COVID-19: one in an Italian woman after treatment with dexamethasone and tocilizumab [51], and another in a 68-year-old man who demonstrated disseminated infection after tocilizumab and methylprednisolone treatment [52]. In highly exposed regions, individuals with COVID-19 should be screened for Strongyloides infection before treatment. One possible solution for at-risk patients is a combination of serological testing and the use of ivermectin as a preventive strategy [53].

Lymphatic filariasis is considered a leading cause of infirmity, permanent disability and chronic morbidity, resulting in societal stigma. The early stages are characterized by fever, coughing, chest pain and lymphangitis, and massive blood eosinophilia and leukocytosis are present. Similarly, tropical pulmonary eosinophilia (TPE), caused by infection with *Wuchereria bancrofti*, is also characterized by coughing, fever, chest pain, body weight loss and eosinophilia [54]. CT imaging of the chest reveals trapped air, mediastinal lymphadenopathy, calcification, and bronchiectasis [55].

Human pulmonary dirofilariasis is caused by *Dirofilaria immitis*. At least 50% of patients are asymptomatic; symptomatic cases present with fever, chill, chest pain, dyspnea and weakness, and sometimes hemoptysis. X-ray images of the chest can reveal lung lesions, with lung cancer in rare cases [56]. The most characteristic finding is the presence of necrotic lung parenchyma, as well as a centrally thrombosed artery containing immature worms surrounded by an inflamed fibrous capsule. Pulmonary dirofilariasis is usually observed on a CT scan as a round or oval-shaped nodule, attached to the pleura, with a predilection for the right lower lobe [57].

Toxocariasis is caused by infection with *Toxocara canis/cati* visceral larva migrans (VLM), with children being particularly at risk [58]. Following infection, the larva penetrates into the intestinal wall and migrates to various organs through the bloodstream. Clinical signs include nausea, loss of appetite, fever, cough, dyspnea, abdominal pain, hepatosplenomegaly and generalized lymph node enlargement [59]. Some cases also present severe eosinophilic pneumonia, which is a common laboratory abnormality [60,61,62]. Chest X-ray imaging may identify localized patchy infiltrates, with bilateral pulmonary nodules being the most common finding. In addition, histopathological examination of lung biopsy specimens typically reveals granulomas with multinucleated giant cells, eosinophils and fibrosis [63]. In the case of covert toxocariasis, recurrent abdominal pain may be also observed [63].

Finally, the consumption of Trichinella larvae, most commonly in infected pork or game, can result in the development of trichinellosis, a zoonotic infection [64]. Symptoms include coughing and dyspnea, caused by the presence of migrating larvae in the lungs; in addition, chest X-ray images can reveal pulmonary patchy infiltrates, together with exaggerated, fuzzy lung markings and hilar enlargement. In addition, leukocytosis, eosinophilia, elevation of aminotransferase, aldolase, LDH and CPK are characteristic signs in blood tests [65]. Abdominal pain, diarrhea, nausea, and vomiting may occur in 2 to 7 days after consumption of raw or undercooked meat and may be the first symptoms of trichinellosis.

## 4. Increased Risk of COVID Infection and Vaccination Efficacy

Parasites can not only cause similar symptoms to COVID-19, but can also exacerbate infection. In this regard, the greatest threat is presented by parasites such as malaria, schistosomiasis and STHs, which cause anemia, pneumonia, neural infections and a strong immune response [4]. Older patients with comorbidities such as high blood pressure, obesity, diabetes and cardiovascular disease, are particularly susceptible to acute COVID-19 disease; however, those with cerebrovascular diseases, chronic obstructive pulmonary disease, chronic kidney disease or tuberculosis are also at risk [66]. Although mortality rates are currently lower in underdeveloped countries, Gutman et al. [67] predict that this is only a temporary state of affairs, as co-infections with parasitic diseases such as malaria may provoke complications with SARS-CoV-2. COVID-19 often results in organ damage caused by an overreaction of the immune system, i.e., a cytokine storm, resulting in the production of a number of immune cells, which then attack the healthy tissue of the lungs and other organs. Such chronic infection shifts the immune system toward type 2 immunity, characterized by the production of interleukin (IL)-4, IL-5, IL-9, and IL-13 [68].

In addition to the cytokine storm, the pathogenesis of COVID-19 is believed to include the so-called bradykinin storm, which may explain many of the symptoms associated with SARS-CoV-2 infection, ranging from loss of the sense of smell and taste to abnormal coagulation. In this case, SARS-CoV-2 infection disrupts both the renin-angiotensin (RAS) and kinin–kallikrein pathways, sending out bradykinin: a peptide that dilates blood vessels and causes them to leak. This process makes it difficult for oxygen to move from the lungs to the blood and then to all the other tissues of the body, which is a common abnormality in COVID-19 patients [69].

Helminth infections are among the most common infectious diseases. Bradbury et al. [2] highlight the possible negative interactions between helminth infection and COVID-19 severity in helminth-endemic regions and note that alterations in the gut microbiome associated with helminth infection appear to have systemic immunomodulatory effects. It has also been proposed that helminth co-infection may increase the morbidity and mortality of COVID-19, because the immune system cannot efficiently respond to the virus [3]; in addition, vaccines will be less effective for these patients, but treatment and prevention of helminth infections might reduce the negative effect of COVID-19. During millennia of parasite-host coevolution helminths evolved mechanisms suppressing the host immune responses, which may mitigate vaccine efficacy and increase severity of other infectious diseases [4]. Helminth-viral co-infections might impair immunity against coronavirus and consequently increase the risk of COVID-19.

In a study based on a series of challenge experiments in animal models immunized with selected coronavirus vaccine candidates, Fonte et al. [5] argue that the impact of helminth infection on both Th1 and Th2 immunity is significant enough be an important consideration in the design and evaluation of vaccines against SARS CoV-2, particularly in helminth-endemic countries. This conclusion is supported by the observed requirements for triggering Th1 responses for controlling viral replication and the development of Th2 immunopathology events [5]: chronic helminth infections were found to stimulate a type 2 immunity response including inter alia IL-4, IL-5, IL-6 and IL-9, leading to the suppression of the immune response against intracellular pathogens. Suppression was found to manifest as increased susceptibility to infectious diseases such as HIV/AIDS or tuberculosis [5]. Hence, SARS-CoV-2 and helminth co-infection could lead to more severe outcomes, especially in a population with either HIV/AIDS, malaria, or tuberculosis.

Complex immunological changes during pregnancy are known to increase susceptibility to infections. It is suggested suggest that pregnant women are more susceptible to serious complications and death from viral infections [70]. Physiological changes in the respiratory system and immune system during the pregnancy period seem to contribute to this greater risk of SARS-C0V-2 infection [70,71]. As discussed by Vale et al. [70], in the first and third trimester, pregnant women are in a pro-inflammatory state; therefore, the SARS-CoV-2-induced cytokine storm may result in a more severe inflammation process. However, it is still difficult to draw absolute conclusions on whether pregnant women are at increased risk of severe consequences of COVID-19 [71]. On the other hand, intestinal parasites, competing for nutrients with the host, can affect the nutritional status of the woman and may cause intestinal inflammation that reduces optimum nutrient absorption [72]. The side effects of parasitic infections in pregnancy that may increase the likelihood of Sars-CoV-2 infection are as follows: anemia, malnutrition and associated adverse pregnancy outcomes; adverse effect of parasitic infections in pregnancy and on maternal health is well-evidenced. However, complex immunological mechanisms at play in helminthic infections during pregnancy need deeper exploration and understanding, especially in relation to viral co-infections.

## 5. The COVID-19 Pandemic in Relation to Antiparasitic Prevention Programs and Parasitological Diagnostics

**Figure 3:**
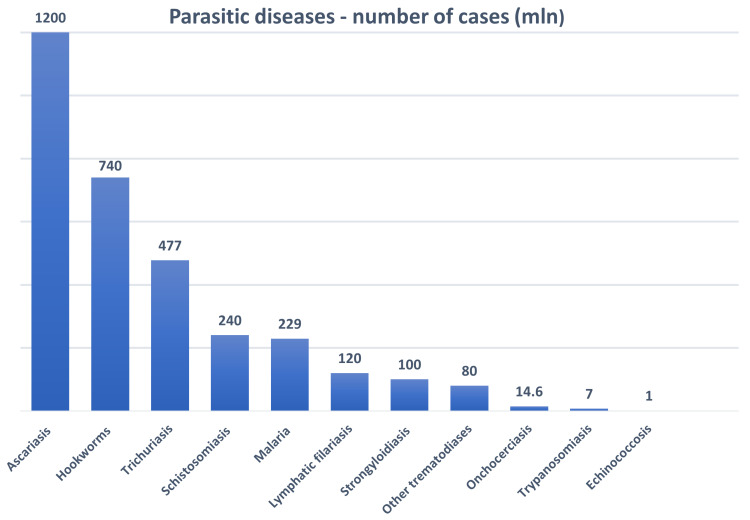
Estimated number of human cases of selected parasitic diseases (source: WHO/CDC).

On 1 April 2020, the WHO issued a general recommendation to interrupt all activities for the control of Neglected Tropical Diseases (NTD) programs, despite the high number of human cases (Figure 3). This has negatively impacted the roadmaps of various NTDs, including those concerning parasitic infections, and has resulted in the loss of many achievements [73]. In addition, parasitic diseases have been largely excluded from the health care tasks at the local, national and regional levels. However, most African countries, especially those where malaria is endemic, demonstrate significantly lower COVID-19 incidence and mortality compared with North America, western Europe or south Asia [30]. The WHO predicts that pandemic-driven shortfalls in prevention and treatment efforts will occur in Sub-Saharan Africa, and that this will result in a higher number of malaria deaths, mostly among children [74]. As the COVID-19 pandemic threatens malaria prevention activities, such as distribution of insecticide-treated nets, indoor residual spraying and malaria chemoprevention, there is a constant need to avoid malaria outbreaks [30]. A 10% disruption in antimalarial programs could lead to 19,000 additional malaria deaths a year, and more plausible disruptions of 25% or 50% are predicted to result in a further 46,000 to 100,000; furthermore, Sherrard-Smith et al. [75] warn that the COVID-19 pandemic could result in an extra 206 million malaria cases and 379,000 deaths in Sub-Saharan Africa.

It is possible that COVID-19 could have set the fight against parasites back by at least a decade, and by implementing lockdowns and restricting the movements of health care providers, these measures could result in more malaria cases and deaths. Additionally, it has been estimated that deaths related to HIV could increase by 10%, tuberculosis by up to 20% and malaria by 36% over the next five years [26]. Most importantly, as the number of COVID-19 related deaths in this region of Africa is still under 40,000, children in Sub-Saharan Africa are more at risk of death by malaria than coronavirus; in addition, due to the closure of schools due to pandemic, over 50 million children have been deprived of free meals, and at least 250 million have been forced out of school, with almost no access to online learning [74].

Two other NTDs whose prevention and control programs were directly impacted by the global and local response efforts to reduce the spread of COVID-19 are soil-transmitted diseases (STDs) and schistosomiasis. Although an interruption in the preventive chemotherapy of STDs will only temporarily impact the progress towards the WHO 2030 target, programs will have to be restarted as soon as possible to minimize the impact on morbidity [76]. After this interruption, additional catch-up time will be required to cope with the higher infection levels.

Importantly, frequent hand washing and disinfection, performed to reduce the number of parasitoses transmitted through dirty hands, will also reduce the chance of SARS-CoV-2 infection. By analogy, restoring programs to prevent, treat and control NTDs, in particular helminths, could well reduce the incidence and mortality of COVID-19 in endemic areas and help to increase vaccination effectiveness.

The COVID-19 pandemic has also had a considerable effect on parasitological diagnostics. As many laboratories are now required to perform SARS-CoV-2 tests, they have had to suspend or reduce their typical parasitological testing duties, which has had a significant impact on the number of diagnosed cases of human parasitoses. The decrease in the number of ordered and performed parasitological diagnostics tests is noticeable in many laboratories. However, we do not yet have accurate and reliable data on this matter.

## 6. Can Parasites Protect Us from the COVID-19?

The low incidence rates of COVID-19 in Africa [74,77,78,79] are of high interest to scientists and WHO authorities, and it has been hypothesized that this could be a result of the increased exposure to parasites in less developed countries: the populations of Africa and Latin America are much more likely to suffer from parasitic diseases than those of more highly developed countries.

Helminth infection entails various forms of immunomodulation, resulting in an increased susceptibility to some infections, a decreased susceptibility to others, and changes in the intensity of allergic, autoimmune and inflammatory diseases; it has also been proposed that infection may account for inadequate responses to vaccines and, possibly, better tolerance of SARS-CoV-2 infection [5,80,81].

The cytokine storm observed in severe cases of COVID-19 [67] is characterized by a predominance of proinflammatory cytokines, such as IL-6. However, it is possible that helminth infection could change the outcome of infection by modifying the Th2 response to limit the inflammatory component [82]; this would be particularly apparent in areas of the world where helminthic infections are still prevalent. Indeed, Ssebambulidde et al. [80] report a lower number of COVID-19 cases in areas where malaria is endemic, schistosomiasis or STH infections; this could suggest a possible protective effect against COVID-19. Interestingly, helminth parasites such as *Fasciola hepatica* have been found to demonstrate immunomodulatory properties, and several Fasciola products have been described as potent immunomodulators [82].

Similarly, an inverse relationship has been reported between the number of cases of certain neglected tropical diseases and those of COVID-19 [73], with one study suggesting that malaria may offer a protective effect against SARS-CoV-2 [30]. Moreover, in patients with SARS-CoV-2 and *Wuchereria bancrofti* co-infection, T cell hypoactivation may cause a relatively milder course of COVID-19 [6].

Additionally, helminth infection may offer promise as part of a protective strategy for other pulmonary diseases, not only COVID-19. Schwartz et al. [83] report that mice with schistosomiasis demonstrated a lower risk of respiratory viral infections, influenza A and murine pneumonia virus, and *Trichinella spiralis* infection was found to limit the inflammatory pulmonary damage induced by influenza virus [84]. Moreover, as discussed by Siles-Lucas, mice infection with *Heligmosomoides polygyrus* and *Trichinella spiralis* may limit immune response against viruses and enhance or reactivate viral infections [82]. These effects have been attributed to the various immunomodulatory and immunosuppressive responses associated with *H. polygyrus* and *T. spiralis* infection, particularly in relation to antiviral Th1 responses; similarly, *Schistosoma mansoni* elicits a biased Th1 response in the early stages of infection [85].

It has been suggested that helminths could enhance antiviral mechanisms, leading to a better control of viral load [5]. During helminth infection, IL-4 can increase and condition virtual memory CD8+ T cells (TVM cells) for more rapid CD8 responses against a subsequent cognate antigen encounter. Most probably, helminth infection has forced the human immune response to evolve a safety mechanism based on the induction of highly responsive TVM cells; this would counterbalance the anti-inflammatory effects related to type 2 immunity and thus result in more effective antiviral responses [5]. The low lethality of COVID-19 in Sub-Saharan Africa may be also related to the inhibition of inflammatory processes by immunomodulatory molecules released by helminths. Indeed, the COVID-19 and Middle East respiratory syndrome-CoV epidemics also caused very limited health problems in the Sub-Saharan region [5].

Additionally, malaria patients develop anti-GPI antibodies that can identify SARS-CoV-2 glycoproteins; these could consequently play a protective role against COVID-19 or ameliorate the disease course [30]. As suggested, in regions where malaria is endemic, hydroxychloroquine and chloroquine prophylaxis may have preventive and/or curative effects against SARS-CoV-2.

Mass preventive chemotherapy of NTDs may result in a lower number of COVID-19 cases in Africa [86]. It is suggested that the low incidence of COVID-19 in Africa may also result from the younger age of the population and possible prior exposure to a cross-reactive viruses. Njenga et al. [79] refute the belief that poor surveillance and low testing numbers are responsible, as reports from local hospitals do not indicate escalating numbers of pneumonia clusters. If the hypothesis does prove to be true, it could be a possible treatment [80].

## 7. Conclusions

Despite being a potentially fatal disease, COVID-19 remains poorly understood. Due to its novelty, it can often be misdiagnosed as other infections affecting the pulmonary system, including parasitic diseases such as malaria, leishmaniasis, shistosomiasis, parogonimiasis, alveococcosis, strongyloidiasis, and trichinellosis. Due to its scale, COVID-19 has had a clear effect on other elimination programs targeting neglected tropical diseases and parasitic disorders. Research indicates that helminth co-infection can have a synergistic or antagonistic effect on the course of COVID-19; however, using the current state of knowledge and the small number of described co-infections, it is difficult to clearly define the influence of parasite infection on COVID-19. Restoring programs to prevent, treat and control NTDs, particularly those associated with helminths, could reduce the incidence and mortality of COVID-19 in endemic areas, and help to increase the effectiveness of vaccination. There is therefore a great practical need for further studies examining the similarities in disease symptoms, as well as the risk factors and the possible role of parasites in the COVID-19 pandemic.

## Data Availability

Statement not applicable as this review did not report new data.
